# The protein acetylase GCN5L1 modulates hepatic fatty acid oxidation activity via acetylation of the mitochondrial β-oxidation enzyme HADHA

**DOI:** 10.1074/jbc.AC118.005462

**Published:** 2018-10-15

**Authors:** Dharendra Thapa, Kaiyuan Wu, Michael W. Stoner, Bingxian Xie, Manling Zhang, Janet R. Manning, Zhongping Lu, Jian H. Li, Yong Chen, Marjan Gucek, Martin P. Playford, Nehal N. Mehta, Daniel Harmon, Robert M. O'Doherty, Michael J. Jurczak, Michael N. Sack, Iain Scott

**Affiliations:** From the ‡ Division of Cardiology,; the § Vascular Medicine Institute, and; the ** Division of Endocrinology, Department of Medicine, and; the ¶ Center for Metabolism and Mitochondrial Medicine, University of Pittsburgh, Pittsburgh, Pennsylvania 15261 and; the ‖ Division of Intramural Research, NHLBI, National Institutes of Health, Bethesda, Maryland 20892

**Keywords:** acetylation, mitochondria, mitochondrial metabolism, fatty acid oxidation, post-translational modification (PTM), GCN5L1, HADHA, liver metabolism, lipid metabolism

## Abstract

Sirtuin 3 (SIRT3) deacetylates and activates several mitochondrial fatty acid oxidation enzymes in the liver. Here, we investigated whether the protein acetylase GCN5 general control of amino acid synthesis 5-like 1 (GCN5L1), previously shown to oppose SIRT3 activity, is involved in the regulation of hepatic fatty acid oxidation. We show that GCN5L1 abundance is significantly up-regulated in response to an acute high-fat diet (HFD). Transgenic GCN5L1 overexpression in the mouse liver increased protein acetylation levels, and proteomic detection of specific lysine residues identified numerous sites that are co-regulated by GCN5L1 and SIRT3. We analyzed several fatty acid oxidation proteins identified by the proteomic screen and found that hyperacetylation of hydroxyacyl-CoA dehydrogenase trifunctional multienzyme complex subunit α (HADHA) correlates with increased GCN5L1 levels. Stable GCN5L1 knockdown in HepG2 cells reduced HADHA acetylation and increased activities of fatty acid oxidation enzymes. Mice with a liver-specific deletion of GCN5L1 were protected from hepatic lipid accumulation following a chronic HFD and did not exhibit hyperacetylation of HADHA compared with WT controls. Finally, we found that GCN5L1-knockout mice lack HADHA that is hyperacetylated at three specific lysine residues (Lys-350, Lys-383, and Lys-406) and that acetylation at these sites is significantly associated with increased HADHA activity. We conclude that GCN5L1-mediated regulation of mitochondrial protein acetylation plays a role in hepatic metabolic homeostasis.

## Introduction

Mitochondrial dysfunction is closely associated with several metabolic disease states and may be a causative or aggravating factor in the development of obesity and type 2 diabetes (1). In the liver, reduced mitochondrial function is a key contributing factor in hepatic lipid accumulation and the development of nonalcoholic fatty liver disease (2). Hepatic fatty acid oxidation is regulated at several biological levels, with defects at the mitochondrial level primarily due to decreased organelle fatty acid uptake (via the carnitine transport system), or by reductions in the activity of mitochondrial β-oxidation pathway enzymes (1, 2).

Excess nutrition leads to an overabundance of available acetyl-CoA, which results in the hyperacetylation of lysine residues on metabolic enzymes (3). In the liver, hyperacetylation of fatty acid oxidation enzymes is typically linked with a decrease in their activity and the subsequent development of metabolic disorders (4–7). To counter this deleterious effect, an enzyme deacetylation system exists in the mitochondria, driven by the sirtuin family protein SIRT3. Using NAD^+^ as a co-factor, SIRT3 catalyzes the removal of acetyl groups from lysine residues, and this activity is essential for the maintenance of mitochondrial metabolic homeostasis (5, 6, 8). However, SIRT3 expression and activity is highly dependent on prevailing dietary conditions, and both are down-regulated in response to excess nutrition (4, 6–7). As such, the protection from mitochondrial dysfunction provided by SIRT3 activity may be limited under nutritional stress.

Research from our group and others has demonstrated that GCN5L1 is required for the efficient acetylation of several mitochondrial proteins and that it functions in direct opposition to SIRT3 *in vitro* (9–11). In this study, we examined whether GCN5L1-mediated lysine acetylation plays a role in the regulation of mitochondrial fatty acid oxidation. Our results show that GCN5L1 is significantly up-regulated in response to a high-fat diet and that transgenic overexpression of GCN5L1 in mouse liver led to increased mitochondrial lysine acetylation. Proteomic analysis identified the fatty acid oxidation enzyme HADHA ^4^ as a putative target of GCN5L1 acetylation, and we show that loss of GCN5L1 reduces HADHA acetylation both *in vitro* and *in vivo*. Finally, we demonstrate that loss of GCN5L1 prevents reductions in HADHA activity in response to a long-term high fat diet. Overall, these data suggest that GCN5L1 plays a key role in the modulation of hepatic fatty acid oxidation under conditions of nutritional excess.

## Results

### Acute exposure to a high-fat diet increases GCN5L1 abundance in the liver

**Figure 1.**
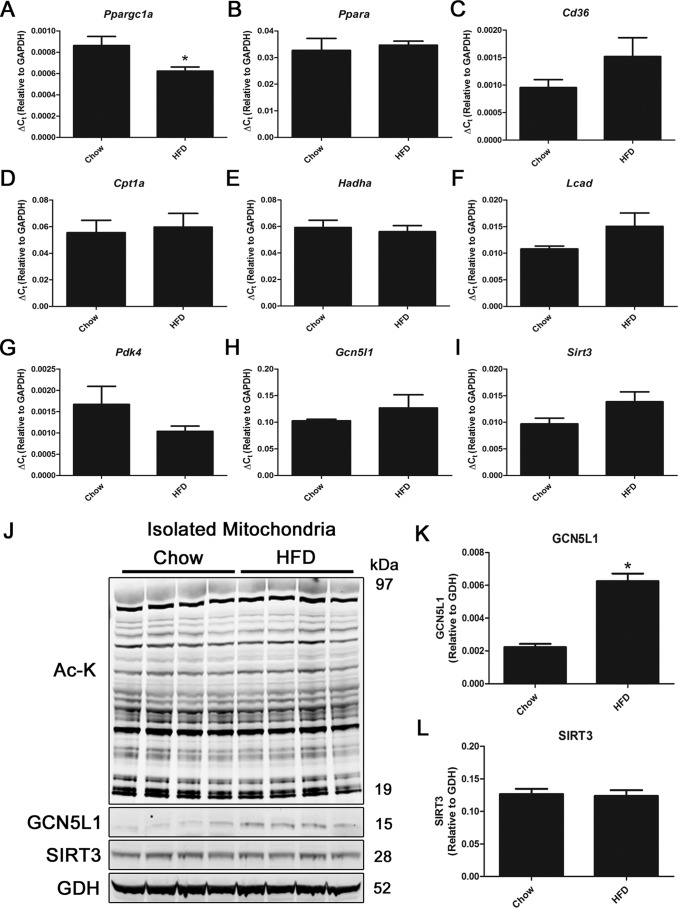
**Short-term HFD exposure increases hepatic GCN5L1 expression.** *A–I*, mice on a HFD for 1 week do not show significant changes in liver mitochondrial metabolic gene expression. *J–L*, although there are no global changes in mitochondrial protein acetylation or SIRT3 expression, there is a significant increase in GCN5L1 protein abundance in the HFD group. *n* = 4; *, *p* < 0.05. *Ac-K*, acetyl-lysine.

We first sought to understand the effect of an acute high-fat diet on liver metabolic regulatory proteins. WT C57BL/6J mice aged 8 weeks were randomly assigned to two groups and either continued to receive a chow diet (10% fat) or were switched to a high-fat diet (60% fat), for 1 week. Gene expression analysis demonstrated a small but significant decrease in *Ppargc1a*, whereas the majority of fatty acid oxidation pathway (*Ppara*, *Cd36*, *Cpt1a*, *Hadha*, and *Lcad*) and glucose utilization (*Pdk4*) genes were unchanged (Fig. 1, *A–G*).

The expression of acetylation regulatory proteins GCN5L1 and SIRT3 remained unchanged at the transcript level following a high-fat diet (Fig. 1, *H* and *I*), which was reflected in a lack of difference in global mitochondrial protein acetylation after 1 week of the diet (Fig. 1*J*). Despite this, there was a 3-fold increase in GCN5L1 protein abundance after 1 week of high-fat diet exposure, which was not matched by changes in SIRT3 (Fig. 1, *J–L*). These data suggest that acute exposure to high fat has a specific effect on GCN5L1 protein abundance in the liver. We therefore designed targeted approaches to model this effect in subsequent studies.

### Transgenic overexpression of GCN5L1 in mouse liver increases mitochondrial protein acetylation

**Figure 2.**
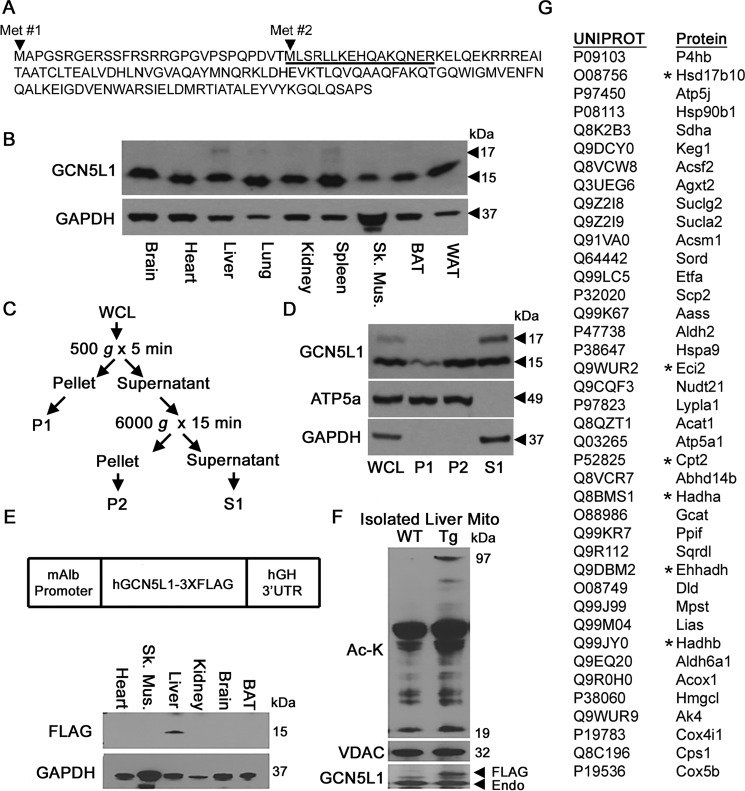
**Liver-specific GCN5L1 Tg mice display elevated mitochondrial protein acetylation.** *A*, sequence and putative start sites of GCN5L1 isoforms. *Underlined sequence* denotes GCN5L1 antibody epitope. *B*, GCN5L1 is present in two isoforms in the liver, lung, and spleen. *C* and *D*, only the 15-kDa protein isoform is present in mitochondria (P1 fraction). *E* and *F*, mice overexpressing mitochondrial GCN5L1 under the albumin promoter display increased mitochondrial protein acetylation in the liver. *G*, proteins that share elevated hepatic acetylation levels at a specific identified lysine residue in both GCN5L1 Tg–overexpressing and SIRT3 knockout mice. *, fatty acid oxidation–related protein. *WCL*, whole-cell lysate.

Analysis of the GCN5L1 sequence revealed the existence of two potential transcriptional start sites, resulting in methionine residues at sites 1 and 29 of the deduced protein sequence (Fig. 2*A*). We therefore examined which of these potential isoforms may be of relevance to hepatic mitochondrial function. Protein expression analysis from multiple tissues revealed that the shorter ∼15-kDa protein was the major isoform in all tissues, with the larger ∼17-kDa protein detectable in trace amounts in a few tissues, such as liver and spleen (Fig. 2*B*). To test whether the shorter isoform was the result of proteolytic cleavage (*e.g.* removal of a targeting sequence upon import to the mitochondria (12)) as opposed to altered transcriptional initiation, we performed subcellular fractionation of liver tissue into different cellular compartments. We found that the shorter GCN5L1 isoform was associated with both the mitochondrial/heavy membrane (P2) fraction and the soluble cytosolic (S1) fraction, indicating that it is not the result of mitochondrial protease activity (Fig. 2, *C* and *D*).

As the shorter GCN5L1 isoform was the sole mitochondrial resident, we focused on this protein in our subsequent experiments. To phenocopy the increased abundance of GCN5L1 in the liver of mice exposed to an acute high-fat diet, we created a transgenic (Tg) mouse line where GCN5L1 was expressed at moderately increased levels under a liver-specific promoter (Fig. 2*E*). Analysis of global mitochondrial protein acetylation by Western blotting showed that GCN5L1 Tg mice had elevated acetylation levels at multiple molecular weights relative to WT controls (Fig. 2*F*). The GCN5L1 Tg mice were not viable after two generations (data not shown), suggesting that constitutive overexpression of GCN5L1 in the liver is detrimental. Despite this, tissue from extant Tg mice was harvested and used to understand how GCN5L1 overexpression affected mitochondrial lysine acetylation more precisely, using proteomic techniques to identify sites that were significantly hyperacetylated at the same lysine residues as those found in SIRT3 knockout mice. Our analysis identified 40 proteins with lysine residues that are predicted to be co-regulated by GCN5L1 and SIRT3 (Fig. 2*G*). Of these, 15% are involved in the mitochondrial fatty acid oxidation pathway, and we focused on this subset of genes for our biochemical analyses.

### GCN5L1 interacts with HADHA in vivo and is required for its acetylation in vitro

**Figure 3.**
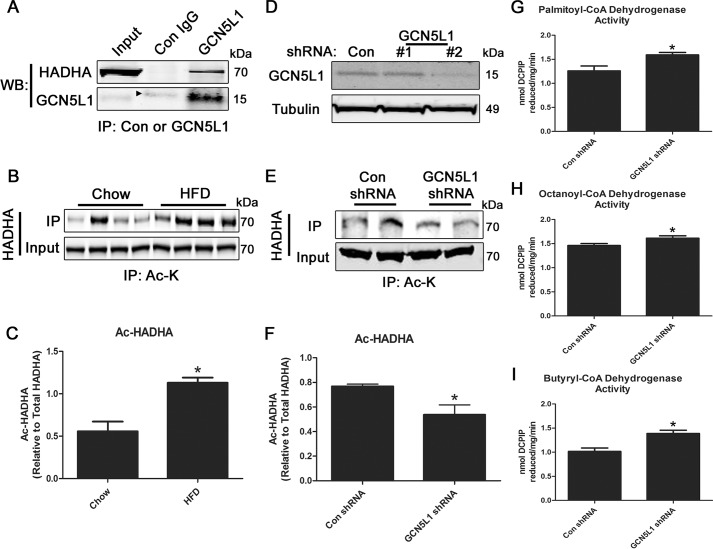
**Liver HADHA acetylation is elevated following short-term HFD exposure.** *A*, HADHA co-immunoprecipitates with GCN5L1 in mouse livers. The *arrow* denotes a nonspecific band at a higher molecular weight in the control IgG fraction. *B* and *C*, HADHA displays elevated levels of lysine acetylation in the livers of mice fed a HFD for 1 week. *D–F*, knockdown of GCN5L1 expression in HepG2 cells significantly decreased HADHA acetylation levels. shRNA line 2 was used for subsequent experiments. *G–I*, loss of GCN5L1 expression increases fatty acid oxidation enzyme activity in HepG2 cell protein lysates. *n* = 4; *, *p* < 0.05. *WB*, Western blotting; IP, immunoprecipitation; *Ac-K*, acetyl-lysine.

To further characterize the potential role played by GCN5L1 in the regulatory acetylation of fatty acid oxidation enzymes, we performed immunoprecipitation experiments to identify GCN5L1 interacting proteins. From this analysis, we found that HADHA co-immunoprecipitated with GCN5L1 in mouse liver tissue (Fig. 3*A*). We next analyzed whether the acetylation status of HADHA was regulated by changes in nutritional status and found that it was significantly hyperacetylated following exposure to high fat for 1 week (Fig. 3, *B* and *C*). Despite being identified in the proteomic screen, the mitochondrial trifunctional protein subunit partner of HADHA, HADHB, showed no changes in nutrient-dependent acetylation (Fig. S1), indicating that HADHA acetylation is more dynamically regulated.

To test whether GCN5L1 may be involved in regulating the acetylation status of HADHA, we genetically depleted GCN5L1 expression in liver HepG2 cells. Stable depletion of GCN5L1 using lentivirus-delivered shRNA was achieved in one cell line (Fig. 3*D*), and this line was used in subsequent experiments. Using immunoprecipitation techniques, we found that HADHA was significantly less acetylated in HepG2 cells displaying reduced GCN5L1 expression (Fig. 3, *E* and *F*). To examine whether this might affect fatty acid oxidation activity, we analyzed the ability of control and GCN5L1-knockdown cells to utilize fatty acyl-CoA species *in vitro*. GCN5L1-depleted HepG2 cells had significantly up-regulated dehydrogenase activity against palmitoyl-CoA, octanoyl-CoA, and butyryl-CoA compared with control cells (Fig. 3, *G–I*), suggesting that reduced GCN5L1 activity may improve hepatic fatty acid oxidation capacity.

### Liver-specific deletion of GCN5L1 protects mice from hepatic lipid accumulation and loss of HADHA activity following a chronic high-fat diet

**Figure 4.**
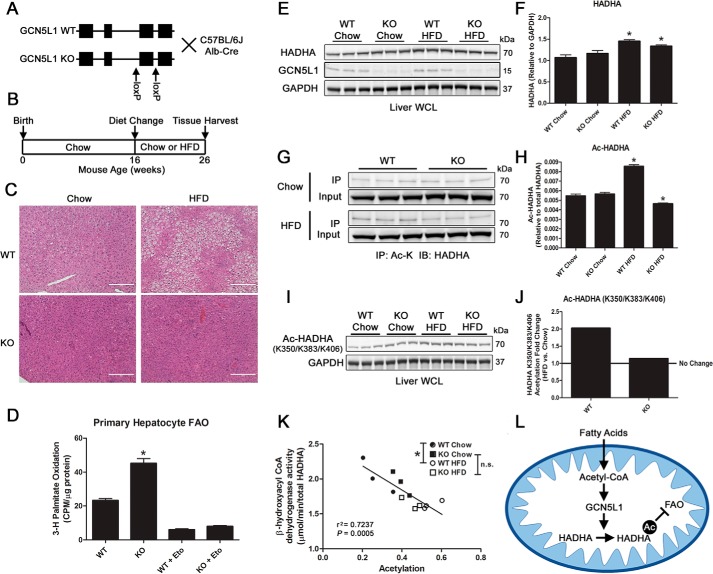
**GCN5L1 liver-specific KO mice are protected from diet-induced hepatosteatosis and loss of HADHA activity.** *A*, GCN5L1 KO strategy. *B*, 16-week-old WT or KO mice raised on a chow diet were randomly placed on either a chow diet or HFD for 10 weeks. *C*, WT mice display elevated hepatic lipid deposition in hematoxylin and eosin staining in response to a 10-week HFD, which is absent in GCN5L1 liver KO mice. *Scale bar*, 200 μm. *D*, GCN5L1 KO primary hepatocytes show significantly increased palmitate oxidation rates relative to WT. These changes are lost when mitochondrial fatty acid oxidation (*FAO*) is blocked with 200 μm etomoxir (*Eto*). *E* and *F*, although elevated in response to a 10-week HFD, there is no significant difference in HADHA expression between WT and GCN5L1 KO mice. *G* and *H*, HADHA acetylation is significantly increased in WT mice following a HFD challenge; however, this response is not seen in GCN5L1 KO mice. *I* and *J*, acetylation of HADHA at lysine residues Lys-350/Lys-383/Lys-406 assessed by a site-specific antibody shows increases in WT, but not KO, mice following a 10-week HFD. *K*, the enzymatic activity of HADHA is negatively correlated with its acetylation status at the Lys-350/Lys-383/Lys-406 lysine residues. HADHA activity is significantly reduced in WT HFD animals *versus* WT chow, but not in KO HFD *versus* KO chow. *n* = 3; *, *p* < 0.05. *L*, model of HADHA regulation by GCN5L1. Fatty acids imported into mitochondria are converted into acetyl-CoA and may be used by GCN5L1 to acetylate HADHA at residues Lys-350/Lys-383/Lys-406. This acetylation reduces the fatty acid oxidation enzyme activity of HADHA, potentially contributing to hepatosteatosis in WT animals. *IP*, immunoprecipitation; *IB*, immunoblotting; Ac-K, acetyl-lysine.

We recently demonstrated that loss of GCN5L1 in the liver negatively impacts hepatic gluconeogenesis by promoting the proteasomal degradation of the transcription factor FoxO1 (13). Using this same liver-specific knockout (KO) mouse line, we examined whether loss of GCN5L1 impacts hepatic fatty acid oxidation. We placed adult WT and KO mice on either a chow (10% fat) or high-fat diet (60% fat) for 10 weeks (Fig. 4, *A* and *B*), after which liver tissues were harvested. In WT mice, there was a substantial accumulation of hepatic lipids after exposure to a high-fat diet, which was absent in mice lacking GCN5L1 expression in the liver (Fig. 4*C*). Primary hepatocytes isolated from GCN5L1 KO mice had significantly increased basal rates of palmitate oxidation relative to WT cells. This difference was lost on exposure to the CPT1A inhibitor etomoxir, showing that this difference depended on mitochondrial fatty acid oxidation (Fig. 4*D*). We next examined whether changes in HADHA acetylation status might contribute to the altered lipid phenotype in GCN5L1 KO mice. At the protein level, both WT and KO mice increased HADHA expression in response to high-fat diet (Fig. 4, *E* and *F*), mirroring the effects seen on the other mitochondrial fatty acid oxidation enzymes analyzed. We next tested whether global HADHA acetylation status was affected by increased dietary fats and found that WT, but not KO, mice displayed HADHA hyperacetylation in response to the 10-week high-fat diet (Fig. 4, *G* and *H*). In agreement with our acute studies, HADHB acetylation was not significantly different under these conditions (Fig. S2). A previous study by Guo *et al.* (13) had demonstrated that HADHA was acetylated on several lysines and that Lys-350, Lys-383, and Lys-406 were involved in maintaining protein stability. As two of these sites (Lys-350 and Lys-406) were detected in our GCN5L1 Tg proteomics screen (data not shown), we tested whether acetylation at these sites was related to GCN5L1 expression in the liver using a site-specific acetylation antibody (14). In primary hepatocytes, we found a significant reduction in basal HADHA acetylation in GCN5L1 KO cells relative to WT (Fig. S3), which matched our observations in HepG2 knockdown cells. *In vivo*, we found that WT mice displayed a roughly 2-fold increase in HADHA acetylation at Lys-350/Lys-383/Lys-406 in response to a high-fat diet, whereas there was almost no increase detected in the acetylation levels at these sites in KO mice relative to chow controls (Fig. 4, *I* and *J*). Using mitochondria isolated from WT and KO mice in an *in vitro* acetylation assay, we found that the addition of exogenous acetyl-CoA led to a nonsignificant elevation of HADHA Lys-350/Lys-383/Lys-406 acetylation in WT, but not KO, mitochondria (Fig. S4). Finally, we examined whether the acetylation status of HADHA at these sites correlated with HADHA activity *in vitro*. We found that there was a significant negative correlation between acetylation levels and HADHA activity (*r*^2^ = 0.7237, *p* = 0.0005, *n* = 12), with high-fat diet animals generally displaying lower activity relative to HADHA abundance (Fig. 4*K*). Importantly, we found that there was a significant decrease in HADHA activity in WT animals on a high-fat diet relative to chow mice, which was not observed in KO animals under the same conditions (Fig. 4*K*). These findings suggest that increased, GCN5L1-dependent hepatic HADHA acetylation occurs in response to a high-fat diet, which negatively impacts fatty acid oxidation activity (Fig. 4*L*).

## Discussion

Acetylation is one of a growing number of acyl-based post-translational modifications of lysine residues on mitochondrial enzymes, which now includes succinylation, malonylation, glutarylation, and methylglutarylation (3). In a similar manner to the regulation of acetylation by SIRT3, these other lysine acylation events are reversed by two further members of the sirtuin family, SIRT4 and SIRT5 (3). It has been speculated that each of these modifications may by added via nonenzymatic processes in the mitochondria, where co-factor availability, matrix chemistry, and residue charge may act in combination to attach acyl groups to lysine residues without the activity of an acyltransferase enzyme (3). This hypothesis was exemplified by an elegant *in vitro* study by Wagner and Payne (15), which showed that exposing isolated mitochondrial matrix proteins to different concentrations of acyl-CoAs, or changing reaction pH, led to an alteration in the abundance of lysine acylation. In partial opposition to this theory, our group and others have shown that loss of GCN5L1 prevents the acetylation of several mitochondrial proteins in various tissues, including liver and heart, and in murine embryonic stem cells (9, 11, 16–18). Of particular note, siRNA-mediated depletion of GCN5L1 in liver HepG2 cells largely prevented the acetylation of isolated mitochondrial proteins exposed to exogenous acetyl-CoA, using a technique essentially identical to that in the Wagner and Payne study (9, 15). These results, combined with the current study, suggest that GCN5L1 does indeed play a regulatory role in mitochondrial acetylation. However, the exact mechanism of this role is still uncertain, as GCN5L1 contains only partial homology to the canonical acetyltransferase protein GCN5 (19). Future work will be required to pinpoint its exact role and to define whether it catalyzes the acetyltransferase reaction on its own *in vivo* or whether it plays a role in increasing acetyl-CoA concentrations near lysine residues to produce an acetylation effect through a “semi-enzymatic” process (3).

In the liver, long-chain acyl-CoA dehydrogenase (LCAD) was the first identified fatty acid oxidation substrate of SIRT3 deacetylation, and it was shown that LCAD activity was reduced in SIRT3 knockout mice as a result of LCAD hyperacetylation (5). Further work showed that SIRT3 knockout mice were more susceptible to hepatic lipid accumulation and the development of metabolic disorders, suggesting that hyperacetylation of hepatic fatty acid oxidation enzymes reduced their activity (4–7). Previous work has identified HADHA as a target of lysine acetylation in the liver, with hyperacetylation of three residues (Lys-350, Lys-383, and Lys-406) protecting the enzyme from proteolytic degradation (14). Furthermore, a recent study has shown that HADHA^+/−^ mice display HADHA hyperacetylation and reduced fatty acid oxidation on a high-fat diet. Fatty acid oxidation activity was restored by transgenic SIRT3 overexpression, which also reversed the hepatosteatosis observed in these animals (20). Our study corroborates these findings with regard to HADHA acetylation and activity and suggests that GCN5L1 may act in opposition to SIRT3 to regulate HADHA in the liver. Proteomic studies are now being carried out to determine which lysine residues are co-regulated by these enzymes *in vivo* under nutrient stress conditions, to further understand the site specificity of this process.

Interestingly, the role played by lysine acetylation in regulating fatty acid oxidation may not be consistent between different tissue types. Whereas acetylation of fatty acid enzymes such as LCAD and HADHA typically decreases their enzymatic activity in the liver, this modification has been linked to increased activity in the heart (10–11). At this time, a detailed proteomic assessment of the lysine residues modified by acetylation on LCAD and HADHA in the heart has not been carried out, and this leaves open the possibility that different residues, with opposing effects on enzyme function, are acetylated on the same protein in response to overnutrition in the liver. The biological basis for these differences may make teleological sense in light of the role played by fatty acid oxidation in each tissue. In the liver, fatty acid oxidation is driven primarily by fasting conditions, where extrahepatic lipolysis provides the fatty acids for β-oxidation, which then provides the substrates and bioenergetics for ketogenesis and gluconeogenesis, respectively (21). In contrast, the heart primarily uses fatty acids as a fuel for normal function, and this energy source accounts for ∼70% of the ATP generated for contractile activity (22). As fatty acids provide the majority of free acetyl-CoA for lysine acetylation (23), there would potentially be a block on cardiac function if fatty acid oxidation enzymes were down-regulated by acetylation in the heart. As such, further work will be required to compare the site-specific acetylation of lysine residues on fatty acid oxidation enzymes from different tissues, to better understand the biology behind these processes. Furthermore, these studies will help to elucidate how GCN5L1 function and activity lead to different physiological outcomes in different tissues.

In summary, our findings in this study suggest that GCN5L1 plays a role in the regulation of hepatic fatty acid oxidation. This adds to recent findings showing that GCN5L1 regulates mitochondrial biogenesis and mitophagy (24), hepatic gluconeogenesis via the stabilization of FoxO1 (13), the acetylation of Kif1Bα-binding protein in mouse embryonic stem cells (18), and endosome/lysosome trafficking in neuronal cells (25). These myriad roles indicate that GCN5L1 is involved in vital cell processes, which may be tissue– or cell compartment–specific. As research into GCN5L1 continues, we anticipate further discoveries regarding its role in fundamental biological systems.

## Experimental procedures

### Animal care and use

Animal experiments were approved by the University of Pittsburgh or the NHLBI, National Institutes of Health, Division of Intramural Research institutional animal care and use committees. For long-term feeding studies, GCN5L1 WT or liver-specific KO mice (13) aged 16 weeks were randomly assigned to groups (*n* = 4 per group) and placed on control (70% carbohydrate, 20% protein, 10% fat; Research Diets D12450B) or high-fat (HFD; 20% carbohydrate, 20% protein, 60% fat; Research Diets D12492) diets for 10 weeks prior to sacrifice. For short-term feeding studies, WT C57BL/6J mice (*n* = 4 per group) were placed on control or HFD diets for 1 week prior to sacrifice. To produce liver-specific GCN5L1 transgenic (Tg) mice, the linearized cDNA plasmid for GCN5L1 with a C-terminal 3× FLAG tag was expressed in mice via embryo microinjection under a mouse albumin promoter.

### Protein isolation, Western blotting, and co-immunoprecipitation

For whole protein lysate, tissues were minced and lysed in 1× CHAPS detergent buffer on ice for 1 h. Homogenates were spun at 10,000 × *g*, and the supernatants were collected for Western blotting or co-immunoprecipitation experiments. For subcellular fractionation studies, tissue was lysed on ice in detergent-free sucrose buffer using glass Dounce homogenizers and separated according to Fig. 2*C*.

Protein expression was analyzed using the following primary antibodies: rabbit SIRT3, rabbit acetyl-lysine, rabbit glutamate dehydrogenase, rabbit GAPDH, rabbit VDAC, mouse tubulin, and rabbit pyruvate dehydrogenase (PDH) from Cell Signaling Technologies; rabbit phospho-PDH (Ser-293) from Novus; mouse ATP5a, goat PGC-1a, and rabbit PDK4 from Abcam; mouse FLAG from Sigma; rabbit HADHA, rabbit CPT1a, rabbit LCAD, and rabbit CD36 from ProteinTech; HADHA acetyl-lysine (Lys-350/Lys-383/Lys-406) as reported previously (14); and GCN5L1 as reported previously (9). Fluorescent anti-mouse or anti-rabbit secondary antibodies (red, 700 nm; green, 800 nm) from LI-COR were used to detect expression levels. Protein loading was further confirmed using glutamate dehydrogenase, GAPDH, or tubulin loading controls where appropriate.

For co-immunoprecipitation experiments, protein lysates were harvested in 1× CHAPS buffer, and equal amounts of total protein were incubated overnight at 4 °C with the relevant antibody or an IgG control. Immunocaptured proteins were isolated using Protein G–agarose beads (Cell Signaling Technology), washed multiple times with CHAPS buffer, and then eluted in LDS sample buffer at 95 °C. Protein densitometry was measured using LI-COR software.

### Histology

Livers from chow and HFD mice were excised following euthanasia, fixed in paraformaldehyde, embedded in paraffin, and stained with hematoxylin and eosin.

### Proteomics

To identify acetylated lysine residues, liver tissue from GCN5L1 WT, GCN5L1 Tg, SIRT3 WT, and SIRT3 KO mice was subject to in-solution trypsin digestion, acetylated lysine immunoprecipitation, and LC-MS/MS detection as described previously (26).

### RNA isolation and qRT-PCR

For qRT-PCR, mRNA was isolated using a total RNA extraction kit (Qiagen), and cDNA was produced using a first-strand synthesis kit (Invitrogen). Transcript levels were measured using validated gene-specific primers (Qiagen). Each experiment was carried out at least three times, and representative results (Δ*C_t_* method) are shown. *Gapdh* was used as the internal control.

### GCN5L1-knockdown stable cell lines

HepG2 cells (ATCC, Manassas, VA) were transduced (multiplicity of infection = 2) with two different Mission lentiviral shRNA particles (Sigma) targeting GCN5L1 (denoted KD#1 or KD#2), or a scrambled control sequence. Transduced cells were selected using puromycin, and stable cell lines were verified by qRT-PCR and Western blotting for gene knockdown efficiency.

### Enzyme activity assays

To assess the activity of acyl-CoA dehydrogenase enzymes (SCAD, MCAD, and LCAD), homogenized protein samples were incubated with butyryl-CoA, octanoyl-CoA, or palmitoyl-CoA as described previously (11) Briefly, to assay, for example, LCAD activity, 25 μg of protein is incubated with 0.1 m potassium phosphate, 50 μm 2,6-dichlorophenolindophenol, 2 mm phenazine ethosulfate, 0.2 mm *N*-ethylmaleimide, 0.4 mm potassium cyanide, and 0.1% Triton X-100 at 37 °C for 4 min. The reaction was initiated with 60 μm palmitoyl-CoA, and the rate of absorbance change was measured at 600 nm over 5 min. Activities were converted to moles of substrate oxidized/min/mass of protein against a standard curve. To assess HADHA activity, β-hydroxyacyl-CoA dehydrogenase activity was measured as described previously (10).

To measure palmitate oxidation in primary hepatocytes, 5 × 10^5^ cells were cultured in 1 ml of KHB medium (111 mm NaCl, 4.7 mm KCl, 1.25 mm CaCl_2_, 2 mm MgSO_4_, 1.2 mm NaH_2_PO_4_) supplemented with 2.5 mm glucose, 0.5 mm carnitine, 5 mm HEPES with or without 200 μm etomoxir. A total of 0.3 μl of [9,10-^3^H]palmitate and 4.7 μl of BSA were used per 1 ml of cell culture medium. After the incubation for 2.5 h, supernatants were collected, and 3H_2_O was recovered by chloroform-methanol extraction. The rate of β-oxidation was calculated as the difference between oxidation counts in the presence or absence of etomoxir and expressed as cpm/μg of protein.

### Statistics

Means ± S.E. were calculated for all data sets. Data were analyzed using two-tailed Student's *t* tests or one-way analysis of variance (with Tukey post hoc tests) as appropriate. *p* < 0.05 was considered significant.

## Author contributions

D. T., K. W., M. W. S., B. X., M. Z., J. R. M., Z. L., J. H. L., Y. C., M. G., M. P. P., N. N. M., D. H., R. M. O., M. J. J., M. N. S., and I. S. data curation; D. T., K. W., M. W. S., B. X., M. Z., J. R. M., Z. L., J. H. L., Y. C., M. G., M. P. P., N. N. M., D. H., R. M. O., M. J. J., M. N. S., and I. S. formal analysis; M. G., M. P. P., N. N. M., R. M. O., M. J. J., M. N. S., and I. S. supervision; M. P. P., N. N. M., M. N. S., and I. S. conceptualization; M. P. P., N. N. M., M. N. S., and I. S. funding acquisition; M. P. P., N. N. M., M. N. S., and I. S. investigation; M. P. P., N. N. M., M. N. S., and I. S. methodology; M. P. P., N. N. M., M. N. S., and I. S. writing-review and editing; N. N. M. and I. S. resources.

## Supplementary Material

Supporting Information

## Footnotes

4 The abbreviations used are:

HADHA and HADHB: hydroxyacyl-CoA dehydrogenase trifunctional multienzyme complex subunit α and β, respectively
LCAD: SCAD, and MCAD, long-, short-, and medium-chain acyl-CoA dehydrogenase, respectively
KO: knockout
HFD: high-fat diet
GAPDH: glyceraldehyde-3-phosphate dehydrogenase
PDH: pyruvate dehydrogenase
qRT-PCR: quantitative RT-PCR
shRNA: short hairpin RNA
Tg: transgenic.
