# Supplementary material for: The protein acetylase GCN5L1 modulates hepatic fatty acid oxidation activity via acetylation of the mitochondrial β-oxidation enzyme HADHA
Source: J Biol Chem. 2018 Oct 15;293(46):17676–84. doi: 10.1074/jbc.AC118.005462 (PMC6240879; doi:10.1074/jbc.AC118.005462)
Supplement: Supporting Information [file supp_293_46_17676__index.html]

The protein acetylase GCN5L1 modulates hepatic fatty acid oxidation activity via acetylation of the mitochondrial β-oxidation enzyme HADHA — GCN5L1 regulates HADHA acetylation — The protein acetylase GCN5L1 modulates hepatic fatty acid oxidation activity via acetylation of the mitochondrial β-oxidation enzyme HADHA — ACCELERATED COMMUNICATION: GCN5L1 regulates HADHA acetylation — Supporting Information 

# The protein acetylase GCN5L1 modulates hepatic fatty acid oxidation activity via acetylation of the mitochondrial β-oxidation enzyme HADHA

## Supporting Information

- Supporting Information (to be published online) - Four supplemental figures and methods
