# Supplementary material for: The protein acetylase GCN5L1 modulates hepatic fatty acid oxidation activity via acetylation of the mitochondrial β-oxidation enzyme HADHA
Source: J Biol Chem. 2018 Oct 15;293(46):17676–84. doi: 10.1074/jbc.AC118.005462 (PMC6240879; doi:10.1074/jbc.AC118.005462)
Supplement: Supporting Information [file supp_AC118.005462_140250_2_supp_218946_pgnbrm.pdf]

SUPPLEMENTAL EXPERIMENTAL PROCEDURES

In order to understand the substrate-enzyme relation of GCN5L1 and HADHA, an *in vitro* enzymatic assay was performed following the protocol by Wagner et. al. [**15**]. Briefly, mitochondria isolated from GCN5L1 WT and KO mice using the QProteome Mitochondrial Isolation Kit (Qiagen) were resuspended and frozen in a non-denaturing buffer containing 50 mM Tris-Cl (pH 8.0 at 37 ^{o}C) and 150 mM NaCl. Mitochondria were thawed on ice, sonicated for 15 seconds twice and then centrifuged at 12000 x g for 10 minutes at 4 ^{o}C. Supernatants comprising the soluble mitochondrial protein were used for the experiment. 5 mM stock solution of acetyl-CoA was made in 50 mM Tris-Cl (pH 8.0 at 37 ^{o}C) and 150 mM NaCl. The appropriate dilution of acetyl-CoA was added to mitochondrial protein to achieve the desired concentration. The reaction was incubated for 90 mins at 37 ^{o}C at 400 rpm in an Eppendorf thermomixer. Following the incubation, protein expression was analyzed using western blot.

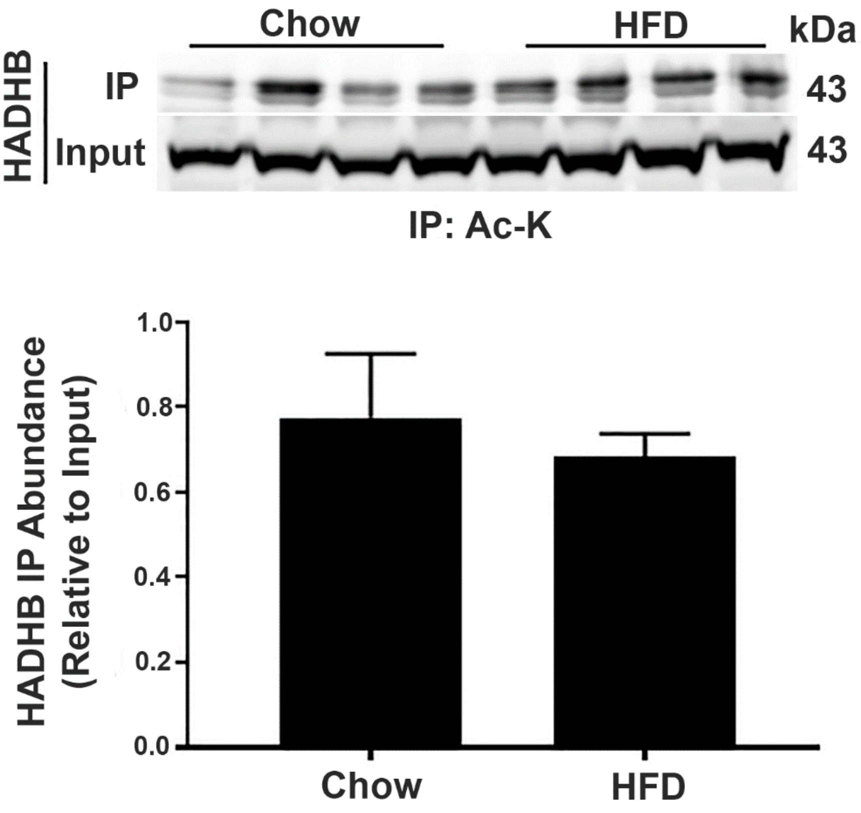

Supplemental Figure 1 – Acetylation of the mitochondrial trifunctional protein subunit HADHB is not responsive to acute changes in nutritional status. Using the same methodology for HADHA (Figure 3), we found no significant changes in HADHB acetylation after 1 week of a high fat diet (HFD). N = 4.

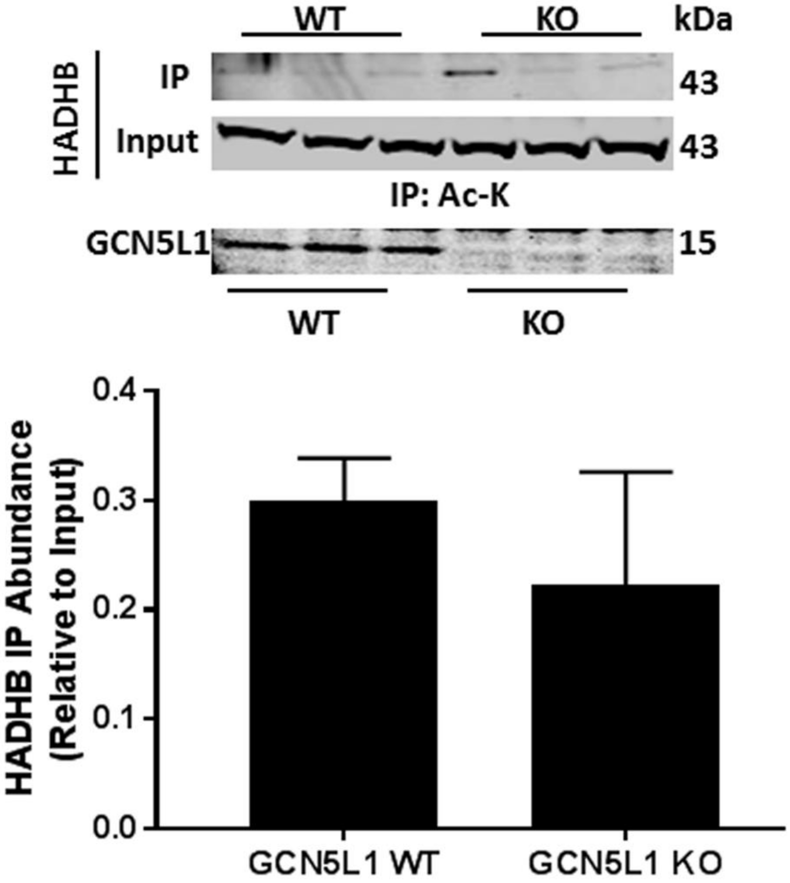

Supplemental Figure 2 – Acetylation of the mitochondrial trifunctional protein subunit HADHB is not responsive to loss of GCN5L1 expression. Using the same methodology for HADHA (Figure 4), we found no significant differences in HADHB acetylation between WT and GCN5L1 KO mice. N = 3.

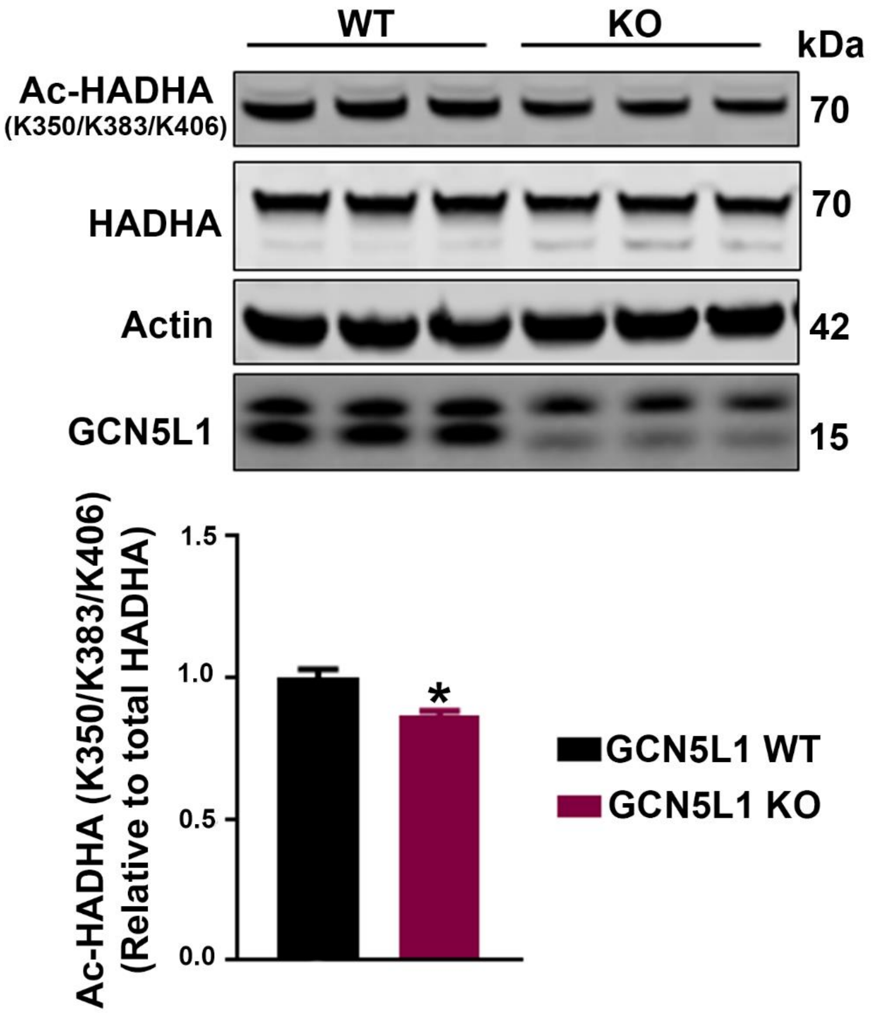

Supplemental Figure 3 – GCN5L1 KO primary hepatocytes display decreased HADHA acetylation at K350/K383/K406. Under basal conditions, primary hepatocytes isolated from GCN5L1 KO mice display less Ac-HADHA levels than WT cells. N = 3.

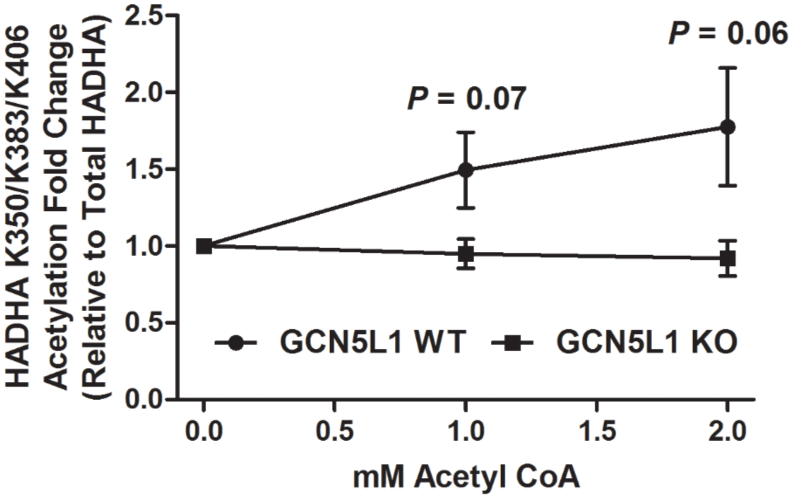

Supplemental Figure 4 – Loss of GCN5L1 expression prevents *in vitro* acetylation of HADHA at K350/K383/K406. Addition of exogenous acetyl-CoA to isolated mitochondria led to a trend towards increased Ac-HADHA in WT mitochondria. This effect was lost in GCN5L1 KO mitochondria. N = 5.
